# Prediction and analysis of COVID-19 daily new cases and cumulative cases: times series forecasting and machine learning models

**DOI:** 10.1186/s12879-022-07472-6

**Published:** 2022-05-25

**Authors:** Yanding Wang, Zehui Yan, Ding Wang, Meitao Yang, Zhiqiang Li, Xinran Gong, Di Wu, Lingling Zhai, Wenyi Zhang, Yong Wang

**Affiliations:** 1 School of Public Health, China Medical University, Shenyang, 110122 China; 2 School of Science, Beijing University of Posts and Telecommunications, Beijing, China; 3 Chinese PLA Center for Disease Control and Prevention, Beijing, 100071 China

**Keywords:** ARIMA, SARIMA, Prophet, COVID-19, Epidemiological dynamics prediction

## Abstract

**Background:**

COVID-19 poses a severe threat to global human health, especially the USA, Brazil, and India cases continue to increase dynamically, which has a far-reaching impact on people's health, social activities, and the local economic situation.

**Methods:**

The study proposed the ARIMA, SARIMA and Prophet models to predict daily new cases and cumulative confirmed cases in the USA, Brazil and India over the next 30 days based on the COVID-19 new confirmed cases and cumulative confirmed cases data set(May 1, 2020, and November 30, 2021) published by the official WHO, Three models were implemented in the R 4.1.1 software with forecast and prophet package. The performance of different models was evaluated by using root mean square error (RMSE), mean absolute error (MAE) and mean absolute percentage error (MAPE).

**Results:**

Through the fitting and prediction of daily new case data, we reveal that the Prophet model has more advantages in the prediction of the COVID-19 of the USA, which could compose data components and capture periodic characteristics when the data changes significantly, while SARIMA is more likely to appear over-fitting in the USA. And the SARIMA model captured a seven-day period hidden in daily COVID-19 new cases from 3 countries. While in the prediction of new cumulative cases, the ARIMA model has a better ability to fit and predict the data with a positive growth trend in different countries(Brazil and India).

**Conclusions:**

This study can shed light on understanding the outbreak trends and give an insight into the epidemiological control of these regions. Further, the prediction of the Prophet model showed sufficient accuracy in the daily COVID-19 new cases of the USA. The ARIMA model is suitable for predicting Brazil and India, which can help take precautions and policy formulation for this epidemic in other countries.

**Supplementary Information:**

The online version contains supplementary material available at 10.1186/s12879-022-07472-6.

## Background

With the widespread of the new coronavirus, it has become a serious threat to the health of people worldwide. This new virus was later named Coronavirus disease 2019(COVID-19), a kind of respiratory infectious disease with lung inflammation [1–3].COVID-19 shows more special transmission characteristics than previous infectious diseases, which leads to its faster transmission speed, wider transmission range, higher transmission risk and rapid epidemic spread, posing a significant threat to global public life and security [4–7].

Since discovering COVID-19 cases in Wuhan, Hubei Province, in December 2019, the epidemic and its variants have spread rapidly throughout the world. So far, the COVID-19 pandemic has spread widely in 188 countries [5], and over 278 million cases and just under 5.4 million deaths have been reported globally 2 according to WHO. At present, the pandemic spread in most countries is still growing and has not been effectively controlled. As of December 28, 2021, the cumulative prevalence of new coronal pneumonia was most outstanding in the USA, followed by India Brazil. Due to the fastest spread of the new coronavirus, the cumulative number of confirmed cases and the daily number of new cases are still increasing in the above countries. Therefore, an analysis of the current cumulative and the new number of cases of COVID-19 has essential research implications for predicting its prevalence trends.

Different models have been used to predict COVID-19 prevalence and mortality rate in recent studies. For example, multiple linear regression [8], Artificial Neural Network [9], multilayer perceptron [10] grey prediction model [11], simulation model [12], Holt model [13], LSTM model [14], and support vector regression [15, 16]. However, the spread of epidemic disease is random and will be affected by many factors [17, 18]. A large number of studies all show that the effect is not best achieved if only a single prediction tool is utilized to predict trends. In addition, the above statistical model can predict the development trend of the epidemic in the medium and long term.

However, as time-series data, COVID-19 cases have some dynamic fluctuation trend in the various situation with epidemic prevention and control [19, 20], which is suitable for establishing a time series model for prediction, but the commonly used single time series analysis model is challenging to capture the nonlinear part of the COVID-19 data series.

Considering that there are many factors affecting the prevalence of COVID-19, these factors lead to the complex characteristics of nonlinear, random and periodic data [21–23], so it is necessary to establish a prediction model and compare the effectiveness of different models. Exploring the prediction effect of the time series and machine learning method on the future of the epidemic can find the potential infectious risk in advance [10], avoid the outbreak of the epidemic, and provide data support for the decision-making of the prevention and treatment of infectious diseases.

The Automatic Regressive Integrated Moving Average (ARIMA) model has some advantages in its simple structure and immediate applicability. The ARIMA model has been applied to the prediction and estimation of prevalent diseases, such as typhoid fever [24], tuberculosis [25], influenza [26] and COVID-19 [27, 28]. Since ARIMA methods do not contain much mathematics or statistics, but also are capable of correlating regulation with short-term changing trends in the time series. So, the model is more suitable for predicting the short-term epidemic diseases. The Prophet is an open-source model that can handle time-series data with the advantages of taking strong seasonal effects, missing data, outliers, and changes in trends [29, 30]. And it is currently useful for predictive analysis of COVID-19. What’s more, the SARIMA and Prophet models can be used to capture some periodic or seasonal changes, further find the nonlinear fluctuations of data, and improve the accuracy of prediction results [31].

**Fig. 1:**
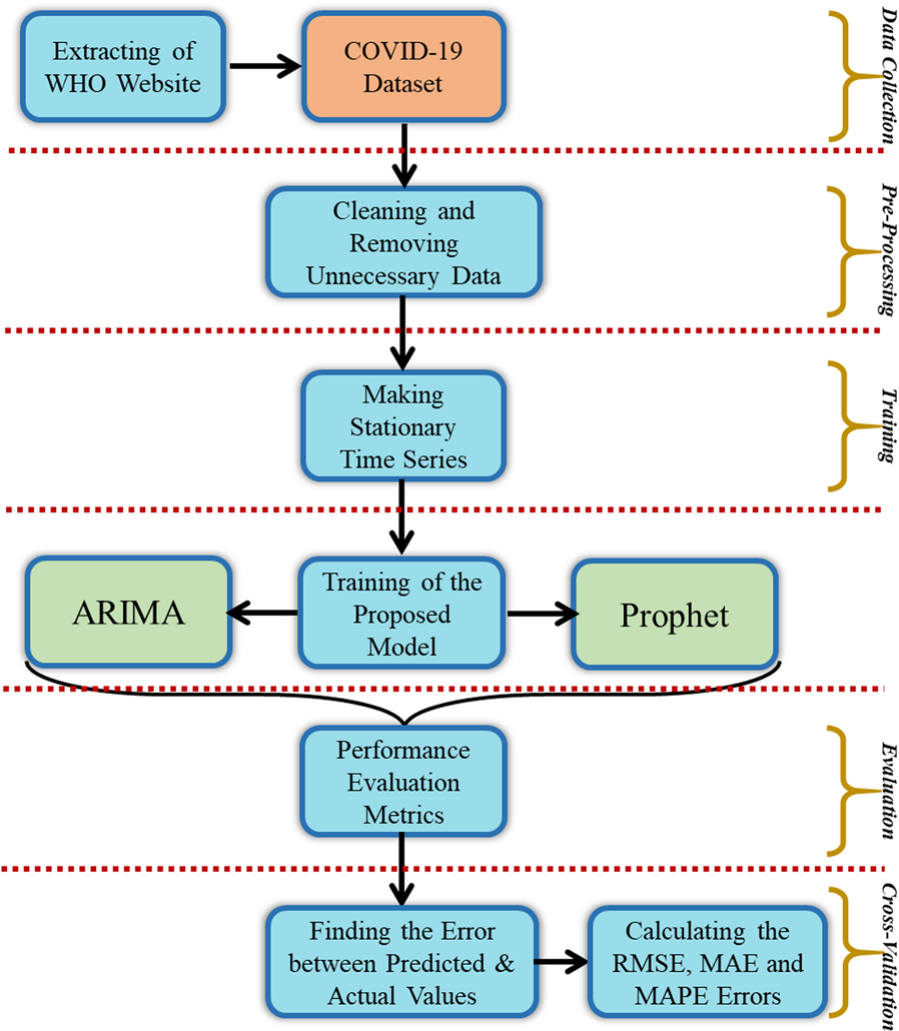
The proposed methodology of the COVID-19 forecasting model

Therefore, it is of great practical significance to predict the daily new cases and cumulative confirmed cases of COVID-19 all over the world. This study establishes the SARIMA, ARIMA and Prophet models to predict the daily new cases and cumulative confirmed cases of COVID-19 in the United States, Brazil and India in the next 30 days ( As shown in Fig. 1), and evaluate the prediction accuracy of the model to provide a further reference for the prediction and early warning of infectious diseases.

## Materials and methods

### Data collection

This article is based on the official WHO website, and MS Excel 2019 was used to build a COVID-19 time-series database. To create a stable and effective ARIMA model, at least 30 observations are required [32]. Cumulative cases and daily confirmed cases from the three countries of the USA, India, and Brazil, as of May 1, 2020, through November 30, 2021, were selected for train data of the construction of disease prediction models and the cumulative cases and daily confirmed cases of those three countries during next 30 days (December 1, 2021, to December 30, 2021) will be forecasted by fitted models. A statistical description of this raw data is presented in Table 1. Forecast the model prediction performance for confirmed case data for the next month with 95% relative confidence intervals (December 1, 2021–December 30, 2021).

**Table 1:** Descriptive statistics on the cases of COVID-19 in USA, Brazil, and India

Cases	Country	Mean	SE Mean	St. Dev	Minimum	Maximum	Skewness	Kurtosis
Cumulative	USA	22,806,184	623,217	14,996,103	1,056,537	48,072,134	− 0.054	− 1.42
	Brazil	10,989,379	309,859	7,455,945	78,162	22,080,906	0.14	− 1.45
	India	15,868,648	521,869	12,557,441	35,043	34,587,822	0.34	− 1.48
New	USA	81,248	2495	60,037	8329	293,310	1.07	0.32
	Brazil	38,012	996	23,975	78,162	22,080,906	0.14	− 1.45
	India	59,680	3329	80,100	1993	414,188	2.81	7.64

### SARIMA and ARIMA model

ARIMA is a type of algorithm for the analysis and forecasting of time series data, namely the Box—Jenkin model, first proposed by Box and Jenkins in the 1970s [32]. The ARIMA (p, d, q) model is known as the differential autoregressive moving average model. Due to the seasonal feature of the raw data, the SARIMA model (seasonal autoregressive integrated moving average), as an extension of ARIMA, is also often used for time series forecasting after seasonal adjustment. Such model is to apply mathematical models to non-stationary time series after smoothing the data, which is used to estimate and extrapolate the state of something at some point in the future by analyzing the pattern of historical data and making future predictions based on that pattern and historical data from the past and the present [33]. The cumulative number of confirmed cases and daily new cases of COVID-19 is a random series with nonlinear or seasonal character, so the model can be considered suitable for forecasting. ARIMA simulates and estimates the state of something at some point in the future. The ARIMA model includes the following steps [34]: Step 1: Assessment of the model; Step 2: The model parameters were estimated; Step 3: Check the hypotheses of the model validation; Step 4: Modeling predictions.The structure of the ARIMA (p, d, q) model is Eq. (1).

1 $${y}_{t}={\varnothing }_{1}{y}_{t-1}+{\varnothing }_{2}{y}_{t-2}+\dots +{\varnothing }_{p}{y}_{t-p}+{e}_{t}-{\theta }_{1}{e}_{t-1}-{\theta }_{2}{e}_{t-2}-\dots -{\theta }_{q}{e}_{t-q}$$

In Eq. (1), ϕ_*a*_(a = 1,2,…,p) and $\theta$_*b*_(b = 0,1,2,…,q) are parameters of the model. y_*t*_ and ɛ_*t*_ represent the original value and arbitrary error at time step t. The arbitrary error represented by ɛ_*t*_ represents σ^2^ with zero mean and standard deviation. Taking the value q = 0 in Eq. (1) works as A.R. model with order p, and for p = 0, it becomes the M.A. model with q order. So (p, q) are both important factors to determine the ARIMA model.

### The Prophet model

The Prophet is a powerful and fast open-source time series model developed by Facebook. which could well handle the impact of missing values and outliers in the time series on the prediction and is suitable for the prediction analysis of the COVID-19 epidemic[35–37]. They are combined in the following equation.

2 $$Y\left(t\right)=g\left(t\right)+s\left(t\right)+h\left(t\right)+{\varepsilon }_{t}$$

where $Y\left(t\right)$ indicates the trend indicator data at time t; $g\left(t\right)$ indicates the trend term and is the portion of the time series in which there is a non-cyclical trend of change; $s\left(t\right)$ indicates the period term and is the portion of the time series that exhibits a periodicity of change; $h\left(t\right)$ indicates a holiday term and is the portion of the sequence that is affected by holidays and, since data from this study do not have an effect of the holiday term in trend projections, this one was not considered; ${\varepsilon }_{t}$ It is an error term which accounts for any unusual changes not accommodated by the model. ${\varepsilon }_{t}$ denotes errors due to unusual changes.

Prophet uses the Fourier series to forecast the seasonality effects, and the seasonality models are specified as the periodic functions of t[38, 39]. The arbitrary smoothing of seasonal effects with a scaling time variable using Fourier series is represented as:

3 $$s\left(t\right)={\sum }_{n=1}^{\infty }{a}_{n}\mathrm{cos}\frac{2n\pi t}{p}+{b}_{n}\mathrm{sin}\frac{2n\pi t}{P}$$

where P is the period and, for a given value of N, to fit the seasonality model, the parameters a1, a2,…, an and b1, b2,…, bn need to be estimated.

### Analytical tools and model evaluation

#### ACF and PACF test

The ACF is a complete autocorrelation function that provides us with the autocorrelation value for any sequence with lag values. In brief, it describes the degree of correlation between the current value of that sequence and its past value. PACF is a partial autocorrelation function. Rather than finding correlations of lags like ACF with the current, it finds correlations of the residuals with the next lag value. An ACF shows the linear relationship between the observations at time t and previous observations at time t − n. The ACF and PACF for a given time series X can be defined as:

4 $$\mathrm{ACF}\left({X}_{t},{X}_{t-n}\right)=\frac{Covariance\left({X}_{t},{X}_{t-n}\right)}{Variance\left({X}_{t}\right)}$$

5 $$\mathrm{PACF}\left({X}_{t},{X}_{t-2}\right)=\frac{Covariance\left({X}_{t},{X}_{t-2}/{X}_{t-1}\right)}{\sqrt{Variance\left({X}_{t}/{X}_{t-1}\right)}\sqrt{Variance\left({X}_{t-2}/{X}_{t-1}\right) }}$$

where in the ACF plot, n is the lag (or difference between ${X}_{t}$ and ${X}_{t-n}$); in the PACF plot between observed values ${X}_{t} and {X}_{t-2}$, n = 2.

#### Performance indices

Three indexes were employed in accessing model fitting and forecasting efficiency: namely Root Mean Square Error (RMSE), Mean Absolute Error (MAE), and Mean Absolute Percentage Error (MAPE), and were applied to test the predictive accuracy of the developed models. Lower RMSE, MAE, and MAPE values indicate a better data fit. The formulations of these criteria are expressed Eqs. (6)–(8), respectively [40]. A logarithmic approach may be necessary to make the time series stationary after differencing. This approach takes the log value of each point, followed by differencing. Bayesian information criterion (BIC) is a class of information criteria to measure the goodness of fit of a statistical model. It builds on the concept of entropy and can weigh the complexity of the estimated model against the goodness of fit of this model to the data. This information helps assess the model's parameters and how well the model performed. In this study, to prevent the excessive model complexity caused by the excessive model accuracy. Therefore, the function sets the lower value.

6 $$\begin{array}{c}RMSE=\sqrt{\frac{SSE}{n}} =\sqrt{\frac{\sum_{i=1}^{n}{\left({Y}_{i}-{\overline{Y} }_{i}\right)}^{2}}{n}}\end{array}$$

7 $$\mathrm{MAE}=\frac{1}{n}\sum_{i=1}^{n}({Y}_{i}-{\overline{Y} }_{i})$$

8 $$\begin{array}{c}MAPE=\frac{100}{n}\times \sum_{i=1}^{n}\left|\frac{\left({Y}_{i}-{\overline{Y} }_{i}\right)}{{Y}_{i}}\right|\end{array}$$

9 $$\mathrm{BIC}=-2\mathrm{log}L\left(\widehat{\theta }\right)+n\mathrm{log}N$$

In Eq. (6), (7), and (8), where ${\mathrm{Y}}_{i}$ is the actual expected output, ${\overline{Y} }_{i}$ Is the model's prediction, i = 1…n and n is the number of observations. In Eq. (9),$\mathrm{log}L\left(\widehat{\theta }\right)$ is the likelihood function, N is the number of observations, and n is the number of model parameters.

### Data analysis

Since the new confirmed cases of COVID-19 has periodically or Seasonal characteristics. The SARIMA model and Prophet model were used to predict next 30 days COVID daily new cases and comfirmed cases data, and the Prophet and SARIMA model were constructed for the prediction of daily cumulative cases. The three models are used for the forecast and simulations of this study based on R 4.1.1 software with forecast and prophet package. Before applying the prediction model, we use logarithmic conversion to process the original data to make the time series more stable and weaken the collinearity of the model, so as to improve the accuracy of prediction.Due to the periodicity of daily new cases, the seasonal components are eliminated,Considering that the daily number of new cases in COVID-19 has the characteristics of periodicity and seasonality,hence, the ARIMA and Prophet model are constructed for the cumulative confirmed case data and, in addition, the SARIMA and prophet model are applied for the daily new confirmed cases.

## Results

### COVID-19 cases description

**Fig. 2:**
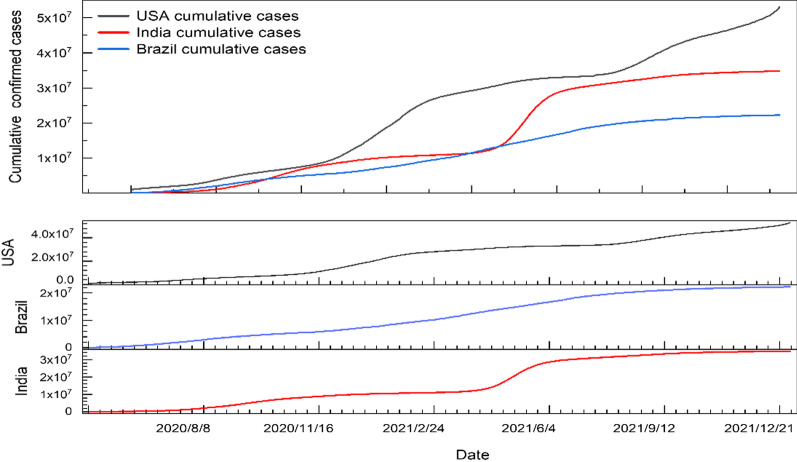
Daily variation of cumulative confirmed cases in USA, Brazil and India from May 1, 2020, to November 30, 2021

**Fig. 3:**
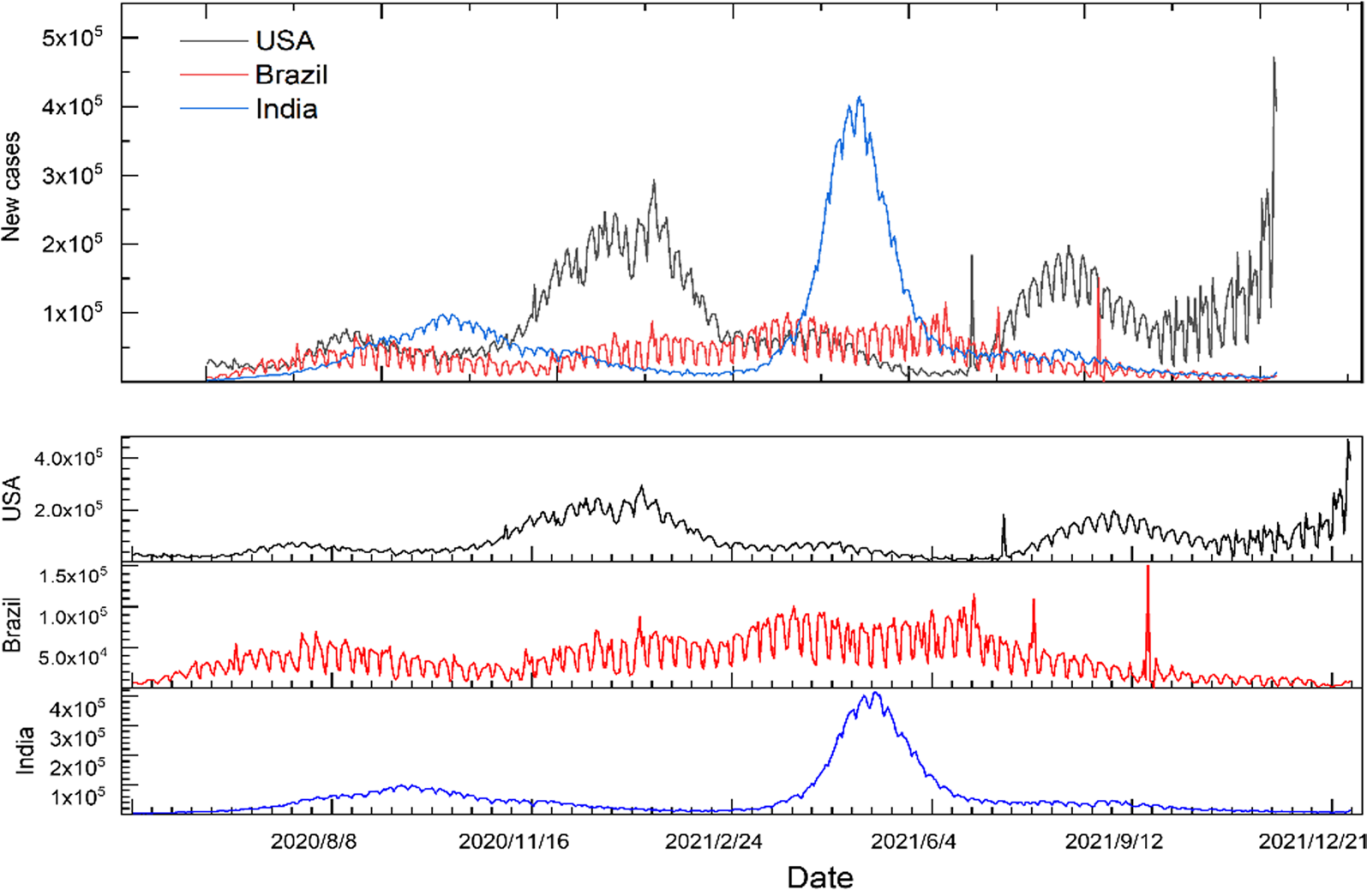
Comparison of daily new confirmed cases in USA, Brazil and India from May 1, 2020, to November 30, 2021

This paper aims to perform a statistical, observational and predictive analysis of a cumulative confirmed case and new confirmed cases dataset from COVID-19 in three countries (USA, Brazil, and India) by ARIMA, SARIMA and prophet model. The COVID-19 data set is divided into 579 days of training samples and the next 30-day of prediction samples (Figs.2, 3). All statistical procedures were performed on the transformed COVID-19 data.

The cumulative number of confirmed cases in the USA is much higher than that in India and Brazil, showing a higher trend than India, with Brazil showing the slowest trend (Fig. 2). The analysis of new cases in three countries shows mainly three peaks of daily new cases in the USA, and they last for a long time. There is a marked peak in daily changes in new cases in India, while the overall trend in new cases in Brazil is relatively stable (Fig. 3).

The increasing trend of cumulative confirmed cases and new confirmed case data could provide more evidence to help citizens understand the time-series variation of COVID-19 in various countries. In addition, the daily new confirmed cases are different from the cumulative confirmed cases with a positive growth trend, which has unstable periodic fluctuations that increase or decrease and may occur seasonally. So we will specifically deal with this part of the period characteristics through the prophet and SARIMA models to facilitate the follow-up prediction.

### Model construction and training

In constructing the ARIMA and SARIMA models, the autocorrelation coefficient ACF and the partial autocorrelation coefficient PACF are analyzed for the smooth time series, respectively. In addition, the indicator with the lowest BIC value was used as the best parameter for the ARIMA model. The optimal combination of model parameters for each indicator is shown in Table 2.

**Table 2:** Determine the best parameters of the SRIMA and ARIMA model

Data	Model	USA		Brazil		India	
		Model structure	BIC	Model structure	BIC	Model structure	BIC
New Cases	SARIMA	(1,1,3) (1,1,1)7	− 357.014	− 326.582	(1, 1, 3) (1, 1, 2)7	64.822	95.242
		(2, 1, 3) (1, 1, 1)7	− 359.761	− 324.982	(1, 1, 2) (1, 1, 2)7	60.225	94.990
		(1, 1, 2) (1, 1, 1)7	− 356.097	− 330.013	(2, 1, 3) (1, 1, 2)7	63.470	89.543
		(1, 1, 2) (1, 1, 1)7	− 366.670	− 336.238	(2, 1, 2) (1, 1, 2)7	65.230	95.649
	Forecast	(1, 1, 2)(1, 1, 1)7			(2, 1, 3)(1, 1, 2)7		
Cumulative Cases	ARIMA	(1, 2, 1)	− 6055.589	− 6042.515	(1, 2, 1)	− 4553.732	− 4540.659
		(2, 2, 1)	− 6061.634	− 6044.202	(2, 2, 1)	− 4625.366	− 4607.935
		(3, 2, 1)	− 6116.429	− 6094.639	(3, 2, 1)	− 4649.157	− 4627.368
		(5, 2, 1)	− 6176.700	− 6146.195	(5, 2, 1)	− 4821.083	− 4790.578
	Forecast	(5, 2, 1)			(5, 2, 1)		

In the prophet model, because the data may have hidden periodicity, such as daily new case data, we mainly adjust the daily and seasonal cycle parameters and growth trend function to predict better. The data and relative and absolute errors of model fitting or prediction are shown in (Additional file 1).

### Model fitting of COVID-19 cumulative cases and daily new cases

**Fig. 4:**
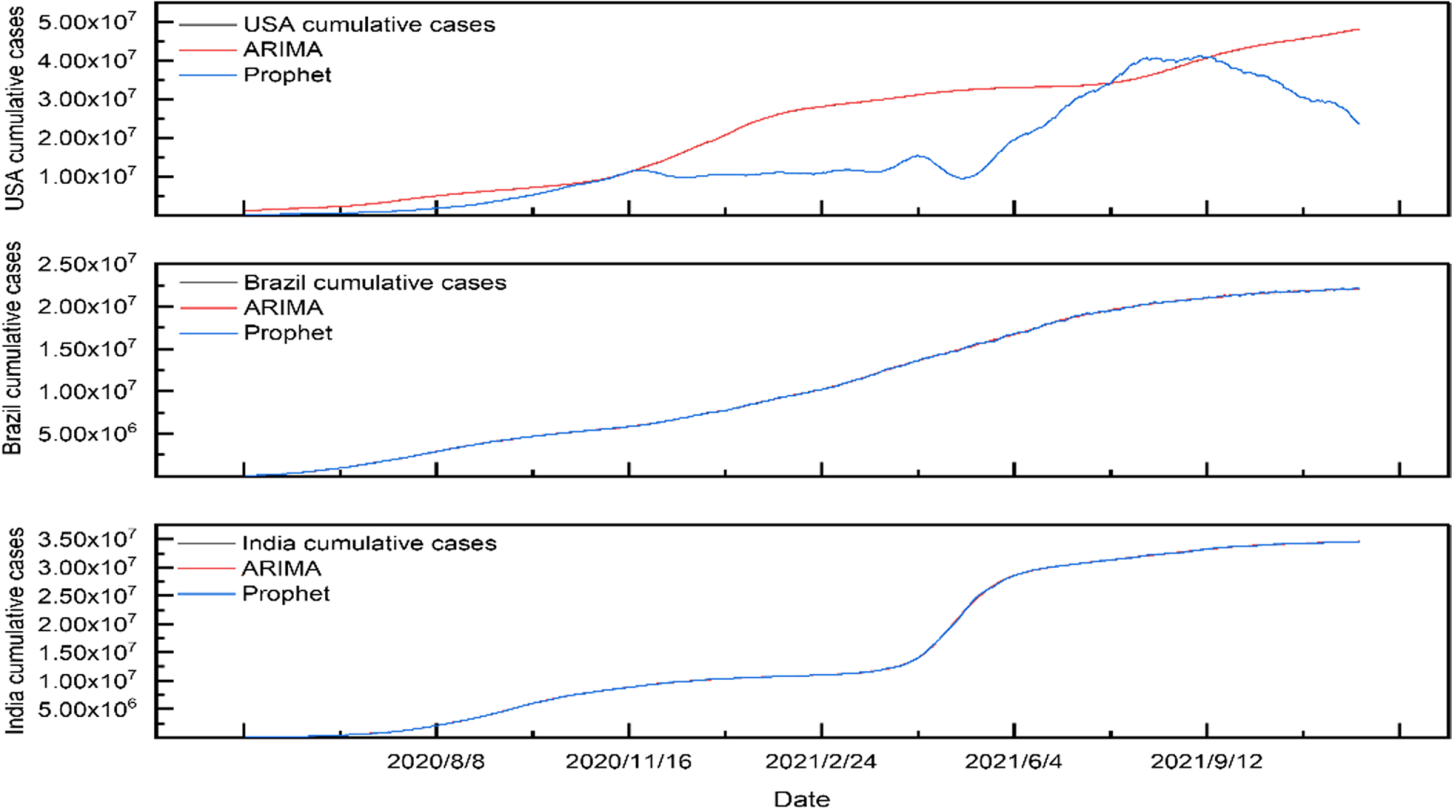
ARIMA and Prophet model fitting for new cumulative cases in USA, Brazil and India from May 1, 2020, to November 30, 2021

Based on the ARIMA and Prophet methods, we trained and studied the statistical data of the cumulative confirmed cases of COVID-19 in the USA, India and Brazil. The learning results are shown in Fig. 4.

In general, when the data fluctuation is slight, these two mos' training and learning results well captured the development trend of confirmed cases with good performance. The fitting results for the cumulative cases of the COVID-19 in the USA show that the average absolute error(MAE) of the ARIMA model is 1847.39, and its mean average relative error(MRE) is 0.00003 is lower than the prophet modle (0.02057). In India, the fitting average absolute error of the ARIMA model is 52.3197 and the mean average relative error is lower than 0.05, and the mean average relative error of Prophet model is also lower than 0.0005 (as shown in Additional file 1). In the training results in Brazil, the average absolute errors are 94.22 (ARIMA) and 2370.45 (Prophet), respectively, and the mean of average relative errors of ARIMA is lower than 0.0005, indicating that the fitting effect is better than that of the Prophet model (0.00001). This result verified that the ARIMA method has a particular advantage in learning the training set of cumulative confirmed cases with slow positive growth.

**Fig. 5:**
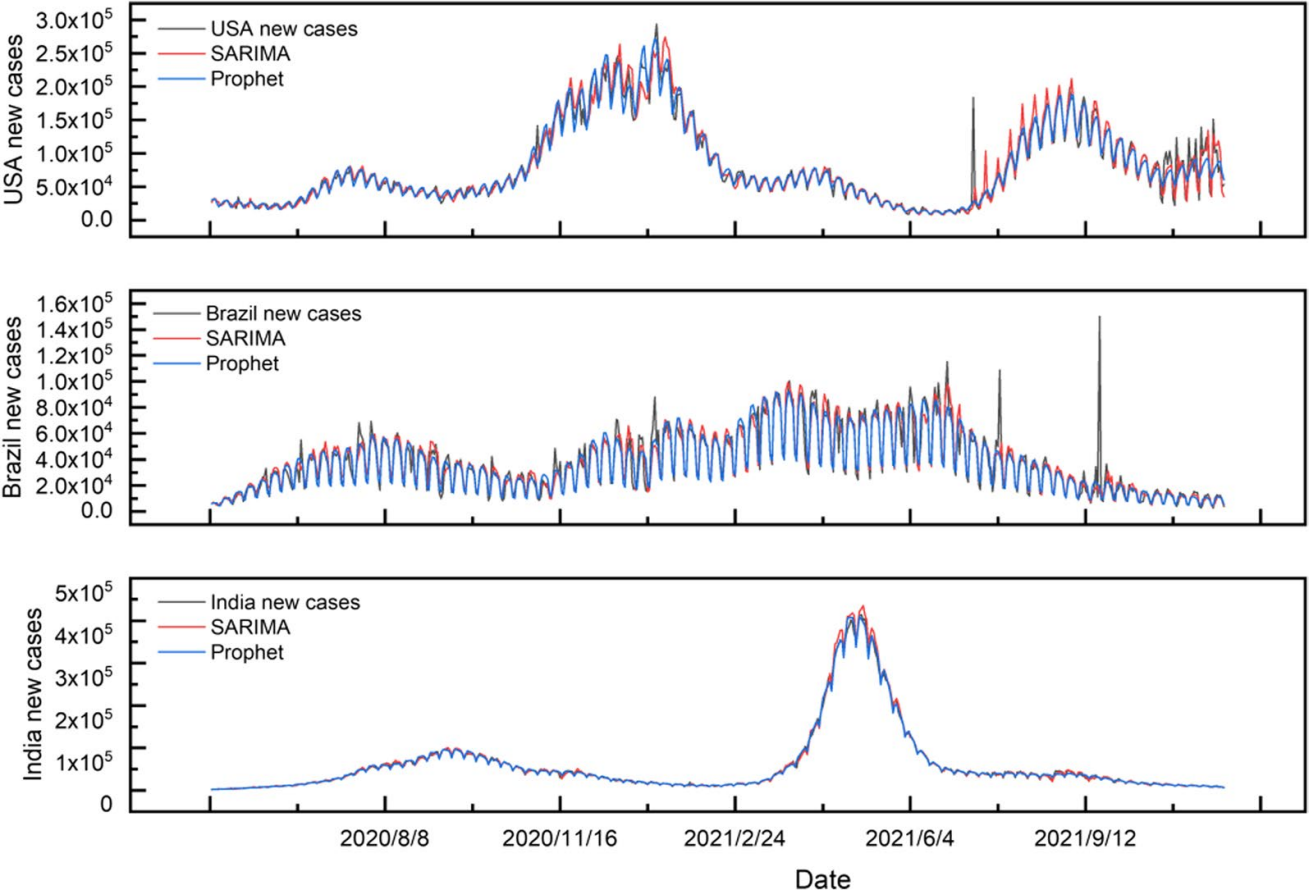
SARIMA and Prophet model fitting for daily new cases in USA, Brazil and India from May 1, 2020, to November 30, 2021

Similarly, we used the SARIMA method and the Prophet method to learn the data of daily new confirmed cases, and the results are shown in Fig. 5. For this kind of fluctuating data, these two methods are better to capture the fluctuation and trend of the data. Both performed better, especially in studying new cases in India (compared to the learning results in the USA and Brazil).

The MAE value of the Prophet model in the USA, India, Brazil is 7118.96, 2331.66 and 4819.42, which is lower than the ARIMA model (7877.08, 2847.32, 5661.42).

This indicates that the effect of the Prophet model is better than that of the SARIMA model in training data. And it is not difficult to find that the Prophet model has more advantages in learning the training time series data with large fluctuations through horizontal comparisons, such as the daily new case.

### Model prediction of COVID-19 cumulative cases and daily new cases

**Fig. 6:**
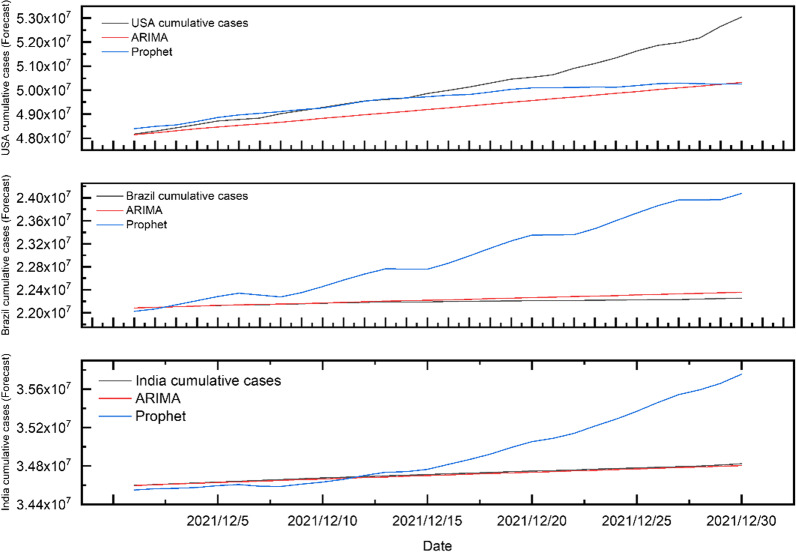
The prediction of the ARIMA and Prophet model for cumulative confirmed cases in the USA, Brazil and India from December 1, 2021, to December 30, 2021

The ARIMA and the Prophet model were used to predict the cumulative confirmed cases of COVID-19 in the next 30 days in three countries, as shown in Fig. 6. Obviously, the prediction trendy for the cumulative cases in the ARIMA model in Brazil and India is more similar to the actual data, which can be in line with the actual trend with a smaller relative error than the Prophet model (Additional file 1).

**Fig. 7:**
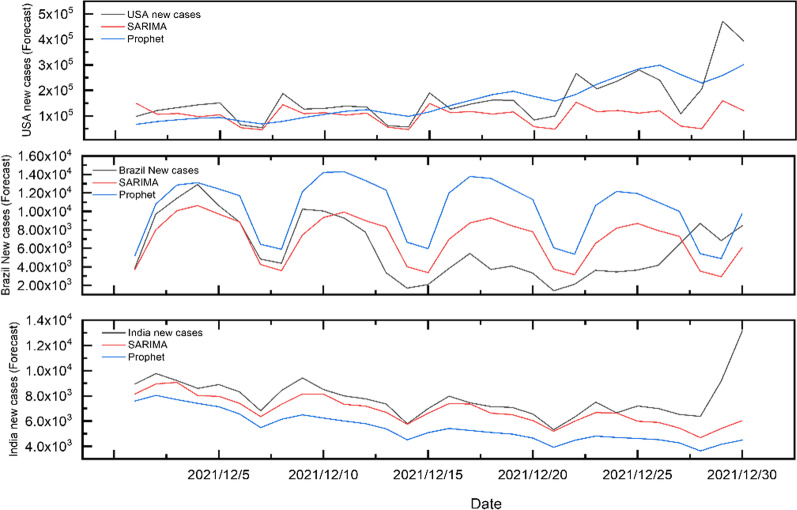
The prediction of the ARIMA and Prophet model for daily new cases in the USA, Brazil and India from December 1, 2021, to December 30, 2021

While in the prediction of new cumulative cases and daily new cases the USA(Fig. 7), the Prophet model is closer to the actual change, unlike the trend in its prediction of daily cumulative COVID-19 confirmed cases and new cases in India and Brazil.

### Accuracy evaluation

It can be seen from Table 3 that the values of RMSE and MAE in the Prophet model for predicting daily new cases and cumulative cases in the USA are lower than those in the SARIMA model. The training model’s R^2^ is similar to the R^2^ value of the SARIMA model, which is basically between 0.8 and 1.0, indicating that the prediction accuracy of the prophet model is higher and can be applied to the actual prediction of COVID-19. In the training dataset for daily new cases, the SARIMA model was over-fitting with poor generalization ability, whereas the Prophet model was fitted the cases and captured the seasonality hidden.

**Table 3:** Accuracy evaluation of ARIMA, SARIMA and Prophet on fitting and forecasting COVID-19 in USA, Brazil and India

	County	Model	Model fitting					Model forecasting			
			R^2^	RMSE	MAE	MAPE		R^2^	RMSE	MAE	MAPE
New Cases	USA	SARIMA	0.942	14,850.734	7877.085	58.421		0.376	100,008.409	67,381.862	10.022
		Prophet	0.950	13,437.603	7118.961	59.665		0.485	67,842.843	50,774.029	10.672
	Brazil	SARIMA	0.821	10,145.700	5661.420	101.147		0.277	2998.022	2490.761	18.477
		Prophet	0.850	9305.905	4819.421	84.732		0.185	5432.666	4593.246	36.437
	India	SARIMA	0.997	5807.807	2847.320	30.662		0.254	1648.759	975.779	3.371
		Prophet	0.997	4073.903	2331.656	29.744		0.244	2697.648	2324.177	8.747
Cumulative Cases	USA	ARIMA	1.000	17,702.819	10,404.341	0.431		0.972	1,149,640.000	903,809.070	0.530
		Prophet	0.745	10,616,818.000	7,862,825.100	242.943		0.084	982,545.500	623,199.520	0.362
	Brazil	ARIMA	1.000	13,046.465	8325.811	1.099		0.949	53,256.662	38,376.992	0.052
		Prophet	1.000	46,232.061	30,472.587	2.072		0.885	985,393.238	784,833.600	1.060
	India	ARIMA	1.000	7057.269	4189.751	0.439		0.999	10,525.591	9883.822	0.009
		Prophet	1.000	43,676.607	26,233.945	1.430		0.880	390,438.575	263,797.980	0.228

The R^2^-value of 1.0 on the graph means that the correlation coefficient is greater than 0.9995, approximately 1.0

But the RMSE, MAE and MAPE values of the cumulative cases were mostly higher in the Prophet model than the ARIMA model in India and Brazil's analysis, which revealed that the ARIMA model has a better ability to fit and predict the COVID-19 cumulative data with a positive growth trend. It should be noted that the fitting and predicted R^2^ value of the model for daily cumulative confirmed cases is close to 1 because its actual value is too large (tens of millions of cases).

## Discussion

With the recent outbreak of a new strain from Omicron, the incidence of a COVID-19 outbreak has reached a new height, increasing the burden on health systems in various countries. But according to the global confirmed case data, the Omicron infection cases have not peaked. According to USA media and health experts, the epidemic situation in the USA maybe even more acute in February. Omicron will continue to ravage through the states of the USA, with a proliferation of fatalities, a severe loss of hospital personnel, a shortage of labor, and several other social and economic confusion in an orderly fashion. The Brazilian Ministry of Health recently announced that multiple cases of Omicron Ba had been diagnosed in the country Cases of BA.2 subtype infection in Omicron. The new epidemic outbreak makes infection control for new strains of coronal pneumonia a tough challenge. Therefore, the construction of the prediction model of the epidemic trend of COVID-19 in most countries provides an essential reference for the prevention and control of the epidemic situation of COVID-19 and provides a decision-making basis for the prevention and control of the COVID-19 epidemic situation in the world.

In the model fitting and prediction stage, in comparing the prediction models between new cases and cumulative cases in different countries, the performance of the two models is better, but there are some differences in various country epidemics.

First of all, the prophet model has a unique advantage in evaluating the daily COVID-19 new cases and has higher accuracy for epidemic data with large fluctuations. This can be explained by The prophet model has better performance than ARIMA in predicting daily new cases and cumulative confirmed cases in the USA, so we can make a preliminary conclusion that when the data fluctuates greatly, its periodic characteristics become more obvious so that the prophet algorithm for decomposition calculation can capture it. This conclusion shows that the Prophet model is based on time series decomposition and machine learning fitting, which can automatically estimate long-term predictions for a wide range of forecasting problems, including marking out possible issues and getting predicted results in a faster time for further investigation by medical staff.

Secondly, in our current work, we have proved a seven-day period in daily new case data from different countries when fitting the SARIMA model. Previous studies used some regression techniques to eliminate time changes in effects and Poisson regression, binomial regression models, normalization, random forests, and other models[41–46]. However, the effect of these models on capturing the accuracy of the seasonal characteristics of events is not stable. In contrast, the analysis results of the SARIMA model are consistent with the actual data trend of COVID-19.

In addition, in this study, the prediction effect of the ARIMA and SARIMA model for India and Brazil with the accurate trend is better than the prophet model, indicating that this model may be more suitable for countries with less fluctuation of epidemic situation. It may be because the daily new cases of COVID-19 are greatly affected by prevention and control strategies and intervention policies leading to the number of cases changes fluctuating significantly, which is not easy to be well-identified by most models.

What's more, the cumulative case data shows a linear change, which can be predicted by some models like ARIMA. It is also why the ARIMA model, as a traditional time series tool, effectively predicts cumulative confirmed cases in three countries. The results further show that the epidemic situation of COVID-19 has a rapidly changing trend, which may be related to the incubation period of the disease and the prevention and control measures taken by the country [references], which is helpful to provide the basis for epidemic prevention and control for health management departments.

## Conclusions

This study introduced the epidemic situation of COVID-19 in the USA, Brazil, and India. The persistent trend and scope of the epidemic were estimated using ARIMA, SARIMA and Prophet models. And found that the Prophet model is more suitable for daily new cases with large fluctuations and has its unique advantages compared with the commonly used ARIMA model. Therefore, we further prove that the three models for the number of cases of COVID-19 can be used to accurately predict the development of infectious diseases and find their potential periodicity. The current work can provide governments regarding make emergency macroeconomic strategies and the allocation of medical resources, regulate social production activities, even give a reference for national economic development, and offer more basis and value for the prediction of COVID-19 epidemics.

## Supplementary Information

**Additional file 1.** The results of predictive model.

## Data Availability

The datasets generated and/or analyzed during the current study are available in the [WHO] repository, https://COVID19.who.int.

## Abbreviations

COVID-19: Coronavirus disease 2019
ARIMA: Automatic regressive integrated moving average
ACF: Autocorrelation function
PACF: Partial autocorrelation function
RMSE: Root mean square error
MAE: Mean absolute error
MAPE: Mean absolute percentage error
